# Novel *Gloeobacterales* spp. from Diverse Environments across the Globe

**DOI:** 10.1128/mSphere.00061-21

**Published:** 2021-07-21

**Authors:** Christen L. Grettenberger

**Affiliations:** a Department of Earth and Planetary Sciences, University of California Davis, Davis, California, USA; University of Wisconsin-Madison

**Keywords:** *Gloeobacter*, evolution, geomicrobiology, photosynthesis

## Abstract

Photosynthetic *Cyanobacteria* and their descendants are the only known organisms capable of oxygenic photosynthesis. Their metabolism permanently changed the Earth’s surface and the evolutionary trajectory of life, but little is known about their evolutionary history. Genomes of the *Gloeobacterales*, an order of deeply divergent photosynthetic *Cyanobacteria*, may hold clues about the evolutionary process. However, there are only three published genomes within this order, and it is difficult to make broad inferences based on such little data. Here, I describe five species within the *Gloeobacterales* retrieved from publicly available databases and examine their photosynthetic gene content and the environments in which *Gloeobacterales* genomes and 16S rRNA gene sequences are found. The *Gloeobacterales* contain reduced photosystems and inhabit cold, wet-rock, and low-light environments. They are likely present in low abundances due to their low growth rate. Future searches for *Gloeobacterales* should target these environments, and samples should be deeply sequenced to capture the low-abundance taxa. Publicly available databases contain undescribed taxa within the *Gloeobacterales*. However, searching through all available data with current methods is computationally expensive. Therefore, new methods must be developed to search for these and other evolutionarily important taxa. Once identified, these novel photosynthetic *Cyanobacteria* will help illuminate the origin and evolution of oxygenic photosynthesis.

**IMPORTANCE** Early branching photosynthetic *Cyanobacteria* such as the *Gloeobacterales* may provide clues into the evolutionary history of oxygenic photosynthesis, but there are few genomes or cultured taxa from this order. Five new metagenome-assembled genomes suggest that members of the *Gloeobacterales* all contain reduced photosystems and lack genes associated with thylakoids and circadian rhythms. Their distribution suggests that they may thrive in environments that are marginal for other species, including wet-rock and cold environments. These traits may aid in the discovery and cultivation of novel species in this clade.

## OBSERVATION

*Gloeobacter* and the newly described sister groups, “*Candidatus* Aurora” and *Anthocerotibacter*, hold important keys to understanding the origin and evolution of oxygenic photosynthesis. These taxa, collectively, the *Gloeobacterales*, are a sister group to the other phototrophic *Cyanobacteria* (non-*Gloeobacterales Cyanobacteria* [NG *Cyanobacteria*]) (1–3). Cultured representatives of this order lack some of the features present in NG photosynthetic *Cyanobacteria*, including thylakoid membranes and several subunits within photosystems II and I (2, 4, 5), and taxa known only from genomic data appear to share these traits (3). Additionally, these taxa have a PsaZ subunit that is not present in NG photosynthetic *Cyanobacteria*. Their PsbO and PsbU subunits have low sequence homology to NG photosynthetic *Cyanobacteria*, and their phycobilisomes are rod-like rather than hemidiscoidal (6). *Gloeobacterales* species have been considered “primitive,” and their traits are inferred to reflect the physiology of early oxygenic phototrophs (3, 7). However, this portion of the cyanobacterial tree is sparsely populated, with only four published taxa, Gloeobacter violaceus, Gloeobacter kilaueensis, *Anthocerotibacter panamensis*, and “*Candidatus* Aurora vandensis.” Therefore, some of these taxa’s unique genetic features may not represent their shared common ancestor and could instead be derived in the species due to their modern environment. In “*Ca.* Aurora vandensis,” the lack of some of these features may result from an incomplete genome rather than absence from the organism itself (3). We need additional species within this area of the phylogenetic tree to determine what characteristics are representative of the clade as a whole so that we can infer the evolutionary history of early phototrophs. These taxa are likely present but overlooked in publicly available data sets, but the abundance of publicly available data makes it difficult to effectively search for these taxa.

**FIG 1:**
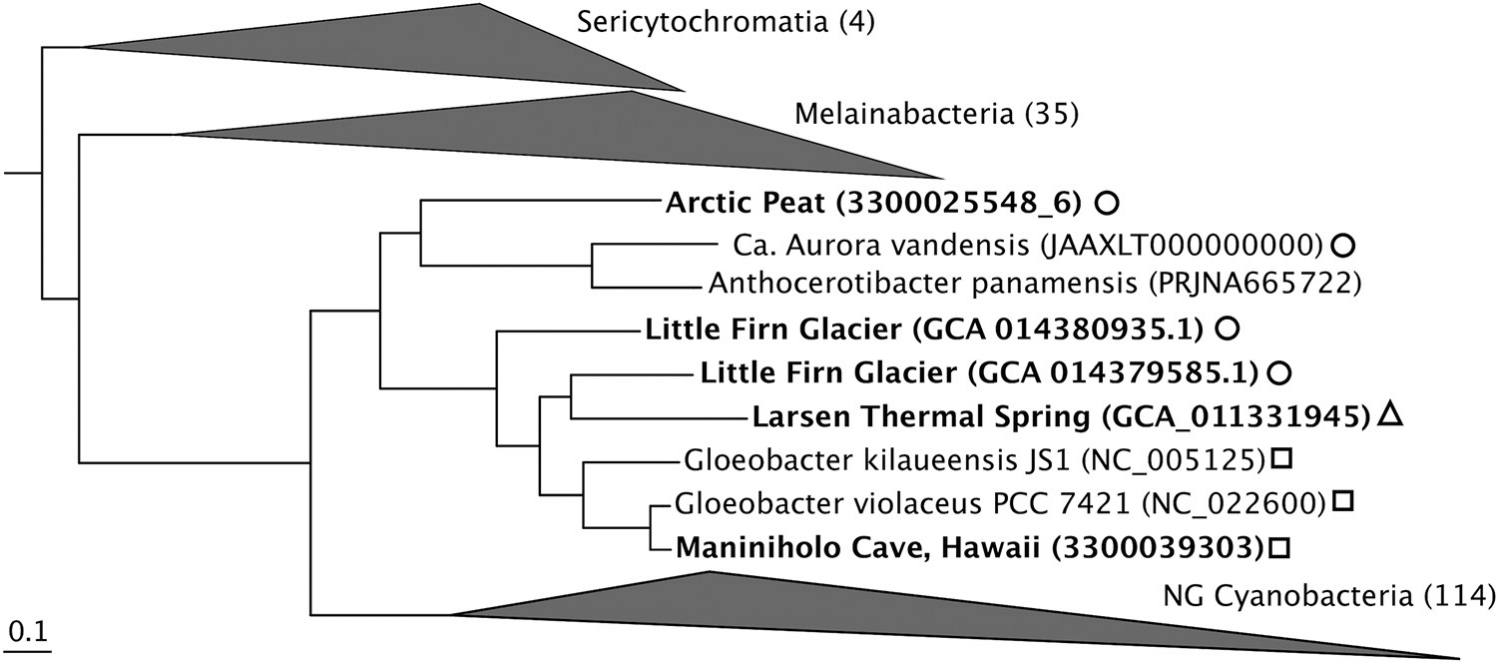
Maximum likelihood tree of 71 concatenated single-copy genes from genomes of “*Candidatus* Sericytochromatia,” “*Candidatus* Melainabacteria,” *Gloeobacterales*, and NG *Cyanobacteria*. New taxa are indicated in bold font. All nodes have 100% bootstrap support. Circles indicate sequences retrieved from alpine, arctic, or Antarctic environments; triangles indicate sequences from thermal springs; squares indicate sequences retrieved from rocky environments.

Five novel taxa of the *Gloeobacterales* were retrieved from publicly available databases (see Text S1 in the supplemental material). Two were retrieved from exposed soil near the Little Firn glacier, a high-Arctic glacier in Greenland (GCA_014379585.1 and GCA_014380935.1), one from Larsen Spring in British Columbia (GCA_011331945.1), one from peat in northern Alaska (GEMs identifier [ID] 3300025548_6), and one from the Maniniholo Cave, Hawaii (IMG genome ID 3300039303_5). These metagenome-assembled genomes (MAGs) are 80% to 99% complete with <1% to 6.5% contamination (see Table S1). The novel and published genomes shared between 92.6% and 69.4% average nucleotide identity (ANI), and four are phylogenetically most closely related to *Gloeobacter* spp., suggesting that they each represent a species within the *Gloeobacter*. The genome from peat is most closely related to “*Ca.* Aurora vandensis” and *A. panamensis* (Fig. 1; Table S1).

10.1128/mSphere.00061-21.1 TABLE S1 Genome statistics, including completeness, contamination, protein-coding genes, and average nucleotide identity with other *Gloeobacterales*. Download Table S1, XLSX file, 0.1 MB. Copyright © 2021 Grettenberger. 2021 Grettenberger https://creativecommons.org/licenses/by/4.0/ This content is distributed under the terms of the Creative Commons Attribution 4.0 International license.

10.1128/mSphere.00061-21.3 TEXT S1 Methodology used for analyses presented here. Download Text S1, DOCX file, 0.1 MB. Copyright © 2021 Grettenberger. 2021 Grettenberger https://creativecommons.org/licenses/by/4.0/ This content is distributed under the terms of the Creative Commons Attribution 4.0 International license.

All of the *Gloeobacterales* genomes lack genes for the PsbY, PsbZ, and Psb27 subunits of photosystem II, the PsaI, PsaJ, and PsaK subunits of photosystem I, PetN, which encodes a small subunit of the B6-f complex, the Kai system that controls circadian clocks, and VIPP1, which plays an important role in building the thylakoid membrane. Some genomes are missing additional photosynthesis-related genes (Table S2), but none of these absences individually would prevent the organism from performing oxygenic photosynthesis (3). The *A. panamensis* and Arctic peat genomes contain two genes absent from the “*Ca.* Aurora vandensis” MAG, PetJ and PsaM. This may indicate that these genes are not absent from “*Ca.* Aurora vandensis” and are the result of genome incompleteness.

10.1128/mSphere.00061-21.2 TABLE S2 Presence and absence of photosynthetic related genes in the *Gloeobacterales* and NG *Cyanobacteria* based on Prokka and GhostKOALA. Genes characteristic of the *Gloeobacterales* (absent from all *Gloeobacterales* or present only in the *Gloeobacterales*) are indicated in green. Genes absent from more than half of the genomes and that may indicate a feature found more broadly in the *Gloeobacterales* are indicated in blue. **, PsaZ was not annotated in *A. panamensis* or “*Ca.* Aurora vandensis,” but a blastp search indicated the presence of sequences with 58% sequence similarity. Download Table S2, XLSX file, 0.1 MB. Copyright © 2021 Grettenberger. 2021 Grettenberger https://creativecommons.org/licenses/by/4.0/ This content is distributed under the terms of the Creative Commons Attribution 4.0 International license.

*G. violaceus* and *G. kilaueensis* are easily inhibited by high light and are slow growing compared to NG *Cyanobacteria* (4), likely due to their reduced photosystems. However, only four species have published genomes, and only three are cultured. Therefore, their traits may not be representative of the clade as a whole. The five new genomes lack the same genes absent from the known *Gloeobacterales*. This suggests that all *Gloeobacterales* species should also be missing these genes and thus be inhibited by light and grow slowly. This slow growth may indicate that these taxa are k-selected species that reduce competition by requiring fewer resources and thrive in environments near their carrying capacity (8).

**FIG 2:**
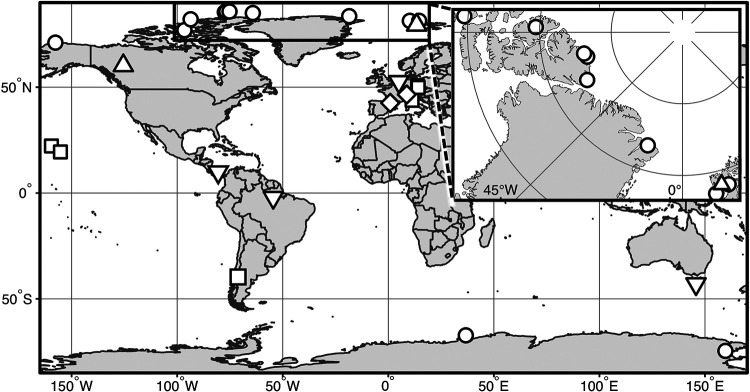
Approximate locations and environments of *Gloeobacterales* based on 16S rRNA gene data and retrieved genomes. Circles indicate sequences retrieved from alpine, arctic, or Antarctic environments; triangles indicate sequences from thermal springs; squares indicate sequences retrieved from rocky environments; diamonds indicate taxa recovered as endoliths in alpine environments; inverted triangles indicate sequences retrieved from other environments. The inset shows locations in the high arctic.

The known environmental distribution of *Gloeobacter* is consistent with them living in environments that are marginal for other cyanobacteria. Indeed, *Gloeobacter* spp. often inhabit wet-rock low-light environments. *Gloeobacter* was first found in limestone rocks in the Alps in the 1970s (6) and then in a lava cave in Kīlauea Caldera, Hawaii (2), a fountain in Florence, Italy (9), and garden waterfalls in Liberec and Teplice, Czech Republic (10). 16S rRNA gene data also showed *Gloeobacterales* in cold climates worldwide (3, 11–15), with additional taxa present in floating pumice (16), lake water and sediments, modern stromatolites (17), and biocrusts or biomass surfaces where they may be protected from light by other biomass (5, 14) (Fig. 2). One of the new genomes comes from Maniniholo Cave, which is likely low light, whereas the light environments for the Larsen Spring and glacial soil samples are unknown.

The environmental distribution of *Gloeobacterales* suggests that it may be productive to search for new taxa in low-light, wet-rock, or cold environments. Additionally, their slow growth and low abundance in some samples (3) indicate that they may be present at low abundance. Therefore, it may be productive to target deeply sequenced data sets that can capture low-abundance taxa.

These five new genomes were found by searching publicly available bins on NCBI and IMG and in the GEMs catalog. Although depositing all bins from a metagenome is becoming more common, most metagenomic data are present either as assemblies on IMG or as raw reads in the Sequence Read Archive. Bins from large numbers of data sets are only available thanks to large well-funded projects like the GEMs project performed at the Joint Genome Institute (JGI) (18). The presence of five new taxa in the small subset of data available as bins suggests that the unassembled and unbinned data likely contain many new taxa in this order and perhaps in closely related undescribed clades. However, reassembling, binning, and searching all available data for these taxa is expensive both in time and computational power. Therefore, we need to develop novel bioinformatics techniques targeted at assemblies or raw reads to facilitate the discovery of novel, early branching cyanobacterial species. Once identified, these novel organisms will help to shed light on the origin and evolution of oxygenic photosynthesis.
