# Supplementary material for: Novel Gloeobacterales spp. from Diverse Environments across the Globe
Source: mSphere. 2021 Jul 21;6(4):e00061-21. doi: 10.1128/mSphere.00061-21 (PMC8386580; doi:10.1128/mSphere.00061-21)
Supplement: TEXT S1 [file msphere.00061-21-s0001.docx]

**Supplemental Methods**

***Genomic Methods.***

I searched the NCBI and IMG databases for taxa classified as Gloeobacterales. Anvi's (v. 6.2) was used to retrieve 71 single copy genes and create a concatenated alignment using MUSCLE (v. 3.8.1551) [1, 2]. I constructed a maximum likelihood tree using RAxML-HPC2 on XSEDE (v. 8.2.12) on the CIPRES Science Gateway using standard parameters including 1000 bootstrap iterations, a Protein GAMMA model, WAG protein substitution matrix, and no correction for Ascertainment bias [3, 4]. The tree contained those genomes identified in our search, the genomes of *G. violaceus*, *G. kilaueensis*, *A. panamensis,* Ca. *A. vandensis*, four Sericytochromatia, 43 Melainabacteria, and 114 non-Gloeobacterales Cyanobacteria. The tree was visualized in Interactive Tree of Life and rooted on the branch containing Sericytochromatia [5]. I used CheckM (v. 1.0.7) to assess the genome quality and used both PROKKA and GhostKOALA to annotate the genomes [6–8]. I compared the presence or absence of the photosynthetic genome content of these genomes to *G. violaceus*, *G. kilaueensis*, *A. panamensis* and *A. vandensis*. Genes were considered present in the genome if they were annotated by either PROKKA or GhostKOALA. PsaZ was not annotated in either *A. panamensis* or Ca. *A. vandensis* but a similar sequence was previously reported in Ca. *A. vandensis* [9]. So I created a custom BLAST database containing the *G. violaceus* PsaA protein sequence and used blastx (v 2.6.0+) to search for similar proteins in these genomes [10].

***Geographic distribution.***

I retrieved all environmental sequences classified as Gloeobacterales in the Arb-Silva database (v. 138)[11] and searched the literature for known reports of taxa within the Gloeobacterales. When possible, I used the metadata in NCBI, IMG or the published papers to determine the environment and location from which each sample was taken. I constructed a map using ggplot2 in R using map data from rnaturalearth (v. 1.0.1) [12] rnaturaldata (v. 1.0.1) [13] and ggOceansMaps (v. 0.4.3) [14].

1. Eren AM, Esen ÖC, Quince C, Vineis JH, Morrison HG, Sogin ML, et al. Anvi’o: an advanced analysis and visualization platform for ‘omics data. *Peerj* 2015; **3**: e1319.

2. Edgar RC. MUSCLE: multiple sequence alignment with high accuracy and high throughput. *Nucleic Acids Res* 2004; **32**: 1792–1797.

3. Stamatakis A. RAxML-VI-HPC: maximum likelihood-based phylogenetic analyses with thousands of taxa and mixed models. *Bioinformatics* 2006; **22**: 2688–2690.

4. Miller MA, Pfeiffer W, Schwartz T. Creating the CIPRES Science Gateway for Inference of Large Phylogenetic Trees. *2010 Gatew Comput Environ Work Gce* 2010; 1–8.

5. Letunic I, Bork P. Interactive tree of life (iTOL) v3: an online tool for the display and annotation of phylogenetic and other trees. *Nucleic Acids Res* 2016; **44**: W242–W245.

6. Seemann T. Prokka: rapid prokaryotic genome annotation. *Bioinformatics* 2014; **30**: 2068–2069.

7. Kanehisa M, Sato Y, Morishima K. BlastKOALA and GhostKOALA: KEGG Tools for Functional Characterization of Genome and Metagenome Sequences. *J Mol Biol* 2016; **428**: 726–731.

8. Parks DH, Imelfort M, Skennerton CT, Hugenholtz P, Tyson GW. CheckM: assessing the quality of microbial genomes recovered from isolates, single cells, and metagenomes. *Genome Res* 2015; **25**: 1043–1055.

9. Grettenberger CL, Sumner DY, Wall K, Brown CT, Eisen JA, Mackey TJ, et al. A phylogenetically novel cyanobacterium most closely related to Gloeobacter. *Isme J* 2020; 1–11.

10. Camacho C, Coulouris G, Avagyan V, Ma N, Papadopoulos J, Bealer K, et al. BLAST+: architecture and applications. *Bmc Bioinformatics* 2009; **10**: 421.

11. Quast C, Pruesse E, Yilmaz P, Gerken J, Schweer T, Yarza P, et al. The SILVA ribosomal RNA gene database project: improved data processing and web-based tools. *Nucleic Acids Res* 2013; **41**: D590–D596.

12. South A. rnaturalearth: World Map Data from Natural Earth. R package  version 0.1.0. 2017.

13. South A. rnaturalearthdata: World Vector Map Data from Natural Earth Used  in “rnaturalearth”. R package version 0.1.0. 2017.

14. Vihtakari M. ggOceanMaps: Plot Data on Oceanographic Maps using “ggplot2”. R package version 0.4.3. 2020.
